# Mode-I Fracture Behavior of CFRPs: Numerical Model of the Experimental Results

**DOI:** 10.3390/ma12030513

**Published:** 2019-02-08

**Authors:** Claudia Barile, Caterina Casavola, Benedetto Gambino, Alessandro Mellone, Marco Spagnolo

**Affiliations:** 1 Dipartimento di Meccanica, Matematica e Management, Politecnico di Bari, viale Japigia 182, 70126 Bari, Italy; casavola@poliba.it; 2 Leonardo Aircraft Spa, Zona ASI Incoronata, 81100 Foggia, Italy; benedetto.gambino@leonardocompany.com; 3 EnginSoft S.p.A., Via A. Murri 2 – Z.I., 72023 Mesagne, Italy; a.mellone@enginsoft.com (A.M.); m.spagnolo@enginsoft.com (M.S.)

**Keywords:** CFRP, delamination test, fracture toughness, numerical modelling, ANSYS Workbench, modeFRONTIER

## Abstract

In the last decades, the increasing use of laminate materials, such as carbon fibre reinforced plastics, in several engineering applications has pushed researchers to deeply investigate their mechanical behavior, especially in consideration of the delamination process, which could affect their performance. The need for improving the capability of the current instruments in predicting some collapse or strength reduction due to hidden damages leads to the necessity to combine numerical models with experimental campaigns. The validation of the numerical models could give useful information about the mechanical response of the materials, providing predictive data about their lifetime. The purpose of the delamination tests is to collect reliable results by monitoring the delamination growth of the simulated in situ cracking and use them to validate the numerical models. In this work, an experimental campaign was carried out on high performance composite laminates with respect to the delamination mode I; subsequently, a numerical model representative of the experimental setup was built. The ANSYS Workbench Suite was used to simulate the delamination phenomena and modeFRONTIER was applied for the numerical/experimental calibration of the constitutive relationship on the basis of the delamination process, whose mechanism was implemented by means of the cohesive zone material (CZM) model.

## 1. Introduction

The use of composite materials has been steadily increasing worldwide in the last decades because of the development of tailoring facilities, allowing for the manufacturing of high performance structures for specific purposes. This characteristic makes such materials highly attractive for applications in different engineering fields in replacement of traditional structural materials such as metals or concrete. It is not unusual to use composite materials for structural applications in aerospace, automotive, and maritime fields, as well as in civil engineering and for sport equipment. Nevertheless, this type of materials is exposed to numerous damage mechanisms that can strictly affect their performance, even in the absence of externally visible damages. This drawback is connected to the presence of technologically different constituent materials, more sensitive to damages with respect to the conventional ones. Interface cracking, that is to say, the loss of cohesion between layers, even known as delamination, is one of the most dangerous and common problems in composite layered materials. The interlaminar deterioration may occur as a result of several reasons, such as low energy impact; high in-service loading; and aggressive conditions, manufacturing defects, or high stress concentration in correspondence with geometrical and/or material discontinuities. This damage mode is particularly critical for structural integrity, because it introduces splitting of the piece into two or more parts, reducing bending stiffness and inducing both local reduction of the strength and the buckling phenomenon, leading to compromised functionality of the parts until they become impracticable in service. Therefore, the quality of the layers’ adhesion strongly influences the safety of a structural layered composite material. The feasibility of fully exploiting the advantages of composite materials requires careful analysis of this critical damage mechanism, as well as a study of the material characteristics corresponding to fracture toughness [1,2]. The intrinsic complexities of the delamination phenomenon due to the singular nature require quantitative assessments of its effects on both the strength and the lifetime of laminated structures, as well as the establishment of universal delamination failure criterions. Analytical efforts and their numerical implementations need to increase their accuracy in order to provide useful predictive instruments to quantify the entity of damages and to estimate the residual lifetime [3]. Experimental data deriving from the delamination fracture tests have to be used for validating the numerical results [4,5,6]. The usefulness of the interlaminar fracture numerical simulation is due to two main reasons. The first one is connected to the replacing of expensive and time-consuming experiments by means of the numerical simulations or virtual testing. For these cases, it is required to reproduce the numerical model in real simplified experimental conditions. The second one relates to the necessity of building innovative analytical models, whose parameters were fitted by comparing the numerical model and the experimental results. Numerical analyses give the possibility to reach goals because they speed up the design process, allowing reliable results. 

The proposed study is related to a research project that involves the development of a structural analysis platform with the aim to perform diagnosis and prognosis in the presence of damaged and/or defected composite material structures. The focus of the research project was the identification, discrimination, classification, and analysis of the most typical composite materials damages/defects experienced in recent years, distinguishing between those that involve materials and those that involve structure components [7,8].

In this paper, a specific class of composite materials is analyzed; it is the well-known class of carbon fiber reinforced plastics (CFRP). CFRPs hold many benefits, such as high value of strength and stiffness. Designing a specific composite material, considering delamination and other forms of internal damages, means to define its damage resistance. This last part represents the aptitude of the material to not collapse at the starting point of damage. The material selection and product development need to be seriously supported by a deep knowledge of the interlaminar resistance of composite laminates [9]. Indeed, the delamination process affects the laminated components in three different modes, indicated as mode I [10,11], mode II [12], and mixed mode [13,14,15]. They have been deeply investigated by traditional experimental techniques and innovative methodologies based on the acoustic emission analysis to attain information about the mechanical behavior of material and the residual lifetime duration of laminated components, in order to increase the accuracy of crack growth monitoring [16,17]. In particular, mode I requires the lowest energy values for the delamination initiation, for this reason, it represents the most known and studied process. Generally, delamination tests are carried out for quantifying the G_I_ fracture toughness of composite material, by following the indication of the ASTM D5528-01 [18]. The calculation considers the energy release rate failure criterion referred to mode I loading condition; it defines the crack growth starting when the G_I_ available energy release rate matches or overcomes the critical value, G_IC_. Precise evaluation of G_IC_ is a crucial phase for better identifying the damage tolerances and the durability of the composites. 

In this work, a numerical model was carried out in addition to the experimental tests performed in accordance with the ASTM D5528-01 procedure [19,20,21]. The finite elements (FE) simulations were a specific topic addressed in this research project owing to the necessity of building the cited analysis platform with diagnosis and prognosis purposes. A specific target of this platform will be the prediction, by means of numerical analyses, of the evolution of the delamination process in the presence of initial defects in composite structural components. In order to accomplish such a goal, experimental tests were used for measuring the fracture energies, necessary to properly calibrate numerical models, representative of the delamination phenomena. Such an approach required, as a first stage, the calibration of a constitutive model characteristic of the delamination in the double cantilever beam (DCB) tests performed on CFRPs. Even if, up to now, academic literature is rich in examples of studies describing DCB tests and their representation by finite element analysis (FEA) [3,4,22,23,24,25,26], this step was mandatory for this research in order to take into account the behavior of the specific constituent materials used, combined with the manufacturing process applied and the related unavoidable data scattering. As far as the FE code is concerned, the choice fell on the ANSYS Workbench Suite commercial one, because it is certified and currently used by the more important end-users (ANSYS Workbench suite is a Multiphysics platform useful to produce high-fidelity virtual prototypes and to simulate the behavior of complete products in their actual working environments). The literature also provides numerous approaches to address this topic (from Virtual Crack Closure Technique to eXtended Finite Element Method [27,28,29,30,31]); in this study, the cohesive zone model (CZM) method implemented on contact elements [32] took privilege. It makes the setup of FE models easier and faster for more complex models (e.g. stringers reinforced panels with initial presence of defects as delamination triggers) by assuming failure mainly as a result of interlayer crack progress [27,30]. The numerical model for the delamination (based on the CZM fracture energy method available in ANSYS) is calibrated on the basis of the experimental data by means of the optimization tool modeFRONTIER (modeFRONTIER is a Multidisciplinary Design Optimization (MDO) platform developed by ESTECO. It is used for streamlining the engineering design process and to cut time and cost while obtaining improved results. It has a workflow-based environment and several multi-objective optimization algorithms).

## 2. Materials and Methods 

### 2.1. Experimental Tests

**Figure 1:**
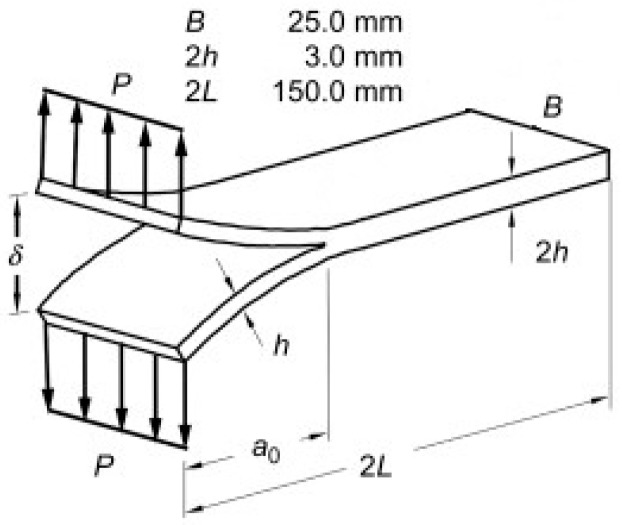
Specimen scheme for load application and dimensions.

**Figure 2:**
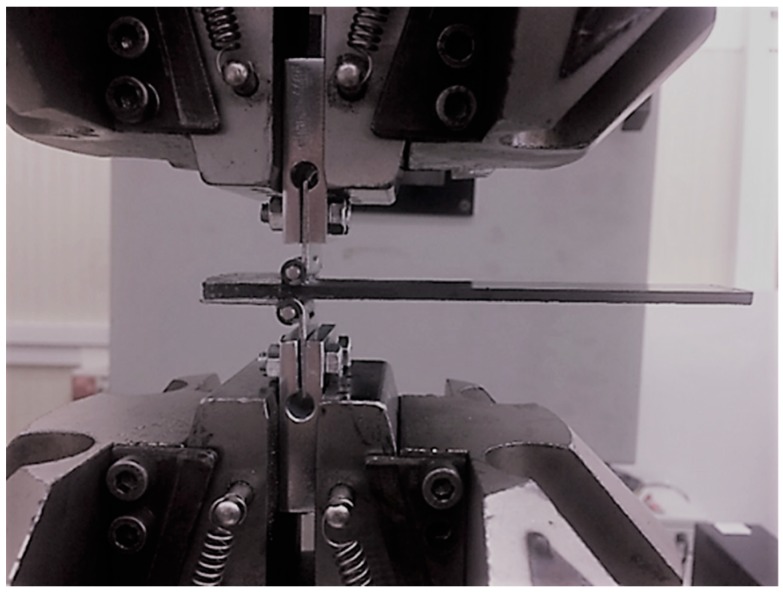
Specimen gripping for mode I delamination tests.

The experimental campaign includes nine CFRP DCB specimens. They were realized by combining carbon fibers and CYCOM® 977-2 resin. The fibers were high performance fibers having both intermediate modulus of elasticity (265-320 GPa) and strength (IMS), the filament diameter was about 5–7 µm, and the resin was a thermosetting one with a density of 1.31 g/cm^3^. Laminates were made by applying a co-bonding process in autoclave curing phase (at 180 °C and at 6 bar of pressure). The half of the plate was firstly cured, then a non-adhesive insert and the not-cured half of the plate were placed on the first part, after which a final curing phase was applied. An even number of unidirectional layers composed the laminates and the delamination growth stirring along the fibers’ direction. A non-adhesive insert was introduced at the midplane of the laminates for inducing the delamination onset [18]. The length of the film was 45 mm, while its thickness was less than 0.013 mm. The specimens were cut from the plates; their sections had a rectangular shape, were 25 mm wide and 125 mm long, and had a uniform thickness of 3 mm (Figure 1). Tests were carried out on a servo hydraulic loading machine, *Instron 1342*, at constant displacement rate (1 mm/min) and at environmental controlled conditions, with T = 23 °C. In Figure 2 the specimen gripped in the testing machine is reported. 

Before each test, a digital grid was realized and calibrated on the specimen. It substituted the manual reference, suggested by the standard, for live monitoring the crack growth. It was superimposed on live recorded images acquired by Charge-Coupled Device camera Marlin AVT. This is a black and white fire-wire camera, having a 1636 × 1252 pixel matrix, running at 12.75 frames per second in this experiment. The entire tests were recorded by placing the optical system in front of each sample; the images were acquired in continuous mode. 

**Figure 3:**
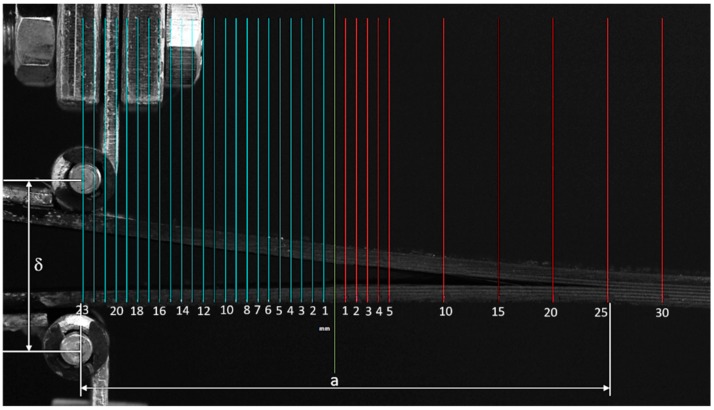
Digital grid for crack growth evaluation.

The blue grid reported in Figure 3, whose length is named a_0_ in the standard calculation, represents the preliminary delamination length included between the pin of the hinge and the end of the non-adhesive insert. The red grid was used for estimating the real crack opening during the test. The total length obtained by combining a_0_ and the real crack opening is defined as a in all the calculation methods.

Different fracture criterions exist for identifying the damage in the materials, and each of them is based on some specific hypothesis. A failure index is evaluated by the stress-based criterions; its critical value is the same as that of the energy-based criterions. Several stress-based criterions (*Tsai-Wu*, *Tsai-Hill*, *Hashin-Rotem, Chang-Chang, etc.*) were proposed [33,34,35]. They were able to distinguish specific failure modes, but their application is not defined in the proximity of crack tips where a singular stress field exists. Griffith was the first to quantitatively connect crack size and strength. Later, Irwin and Rice also seriously contributed to the fracture mechanics [32]. 

The Griffith–Irwin approach is based on linear elastic fracture mechanics. The stress intensity factor or the critical strain energy release rate ruled both the crack initiation and propagation. A remarkable feature is that Griffith’s principle is appropriate to handle the singularity nature of the problem. Griffith’s criterion defines the energy release rate *G* (1) as the modification of the potential energy *U* referring to the crack length *a* [32]:

(1) $$G=-\frac{dU}{B\;da},$$

where a corresponds to the crack length, *B* to the specimen width, and *U* represents the total potential deformation energy of the sample. Crack initiation or propagation may occur if the energy release rate matches the critical value, *G = G*_C_.

Four types of data reduction methods were calculated in all the tests for estimating G_Ic_ values. They consisted of a beam theory method (BT), a modified beam theory (MBT), a compliance calibration method (CC), and a modified compliance calibration method (MCC). However, the MBT method yielded the most conservative values of G_Ic_. 

The BT expression for the strain energy release rate of a perfectly built-in double cantilever beam is as follows:

(2) $$G_{I}^{BT}=\;\frac{3P\delta }{2Ba},$$

where P represents the maximum load and δ represents the deflection in correspondence of the load.

Equation (2) considers a built-in condition in correspondence with the double cantilever beam clamping to the delamination front. The possibility of rotation at the end of the beam, owing to the real clamping conditions, leads to a correction of the previous formulation in favor of the MBT, which provides a new calculation of the strain energy release rate, as follows:

(3) $$G_{I}^{MBT}=\;\frac{3P\delta }{2B\left(a+\;\left|\Delta \right|\right)},$$

where Δ may be experimentally determined by making a least squares plot of the cube root of compliance (C^1/3^), with respect to the delamination length.

The CC method is based on the generation of a least squares plot of log (δ_i_/P_i_) as opposed to log (a_i_), by considering the onset values and the propagation values visually accounted along the delamination. Then, the best least-squares fit was drawn through the data. Finally, the exponent n representing the slope of this line was used to calculate the mode I interlaminar fracture toughness as follows:

(4) $$G_{I}^{CC}=\;\frac{nP\delta }{2Ba}.$$

Lastly, the MCC method was applied. It generates the least squares plot of the delamination length normalized by specimen thickness, a/h, as a function of the cube root of compliance, C^1/3^, by considering the onset values and the propagation values visually accounted along the delamination. The slope of this line, indicated as *A*_1_, was used for evaluating the mode I interlaminar fracture toughness as follows:

(5) $$G_{I}^{MCC}=\;\frac{3P^{2}C^{2/3}}{2A_{1}Bh}.$$

### 2.2. Numerical Model

The numerical model was developed according to the experimental setup by means of the ANSYS Workbench Suite and was based on the cohesive zone material (CZM) model. In detail, the modeling of the composite parts was done by means of ANSYS Composite PrepPost tool. The CZM approach consisted of introducing fracture mechanisms by adopting softening relationships between tractions and separations, which in turn introduced a critical fracture energy that was also the energy required to break apart the interface surfaces [36]. The interface surfaces of the materials can be represented by a special set of interface elements or contact elements, whereas the CZM model can be described as a constitutive relation between the traction T acting on the interface and the corresponding interfacial separation δ (displacement jump across the interface).

**Figure 4:**
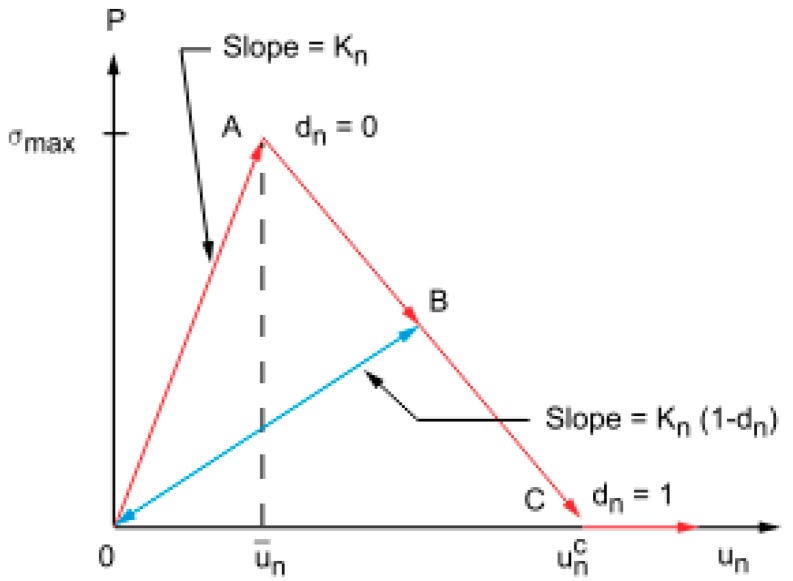
Normal contact stress and contact gap curve for bilinear cohesive zone material.

In particular, the CZM model was based on contact elements, which means that the interfacial separation was defined in terms of contact gap or penetration and tangential slip distance. In particular, cohesive zone modeling with contact elements included two different traction separation laws; namely a bilinear traction separation law and an exponential traction separation law. For this study, the bilinear behaviour with linear softening characterized by maximum traction and critical energy release rate was chosen. According to this behaviour, as described by Alfano [22], mode I debonding defined a mode of separation of the interface surfaces in which the separation normal to the interface dominated the slip tangent to the interface. The normal contact stress (tension) and contact gap behavior are plotted in Figure 4.

Figure 4 shows linear elastic loading (OA) followed by linear softening (AC). The maximum normal contact stress is achieved at point A. Debonding begins at point A and is completed at point C, when the normal contact stress reaches zero value; any further separation occurs without any normal contact stress. The area under curve OAC represents the energy released due to debonding, and is called critical fracture energy. The slope of the line OA determines the contact gap at the maximum normal contact stress and, hence, it characterizes how the normal contact stress decreases with the contact gap, that is, whether the fracture is brittle or ductile. After debonding is initiated, it is assumed to be cumulative, and any unloading and subsequent reloading occurs in a linear elastic way along line OB at a more gradual slope. Following the ANSYS user’s guide [36], the equation for curve OAC can be written as follows:

(6) $$P=K_{n}u_{n}\left(1-d_{n}\right),$$

where *P* is the normal contact stress (tension), *K_n_* is the normal contact stiffness, *u_n_* is the contact gap, and *d_n_* is the debonding parameter.

The debonding parameter for mode I debonding is defined as follows:

(7) $$d_{n}=\left(\frac{u_{n}-{\bar{u}}_{n}}{u_{n}}\right)\left(\frac{u_{n}^{c}}{u_{n}^{c}-{\bar{u}}_{n}}\right),$$

where ${\bar{u}}_{n}$ is the contact gap at the maximum normal contact stress (tension), $u_{n}^{c}$ is the contact gap at the completion of debonding, *d_n_* = 0 for Δ_*n*_ ≤ 1, and 0 < *d_n_* ≤ 1 for Δ_*n*_ > 1 with

(8) $$\Delta_{n}=\frac{u_{n}}{{\bar{u}}_{n}}.$$

The normal critical fracture energy is computed as follows:

(9) $$G_{cn}=\frac{1}{2}\sigma_{max}u_{n}^{c},$$

where *σ_max_* is the maximum normal contact stress.

**Figure 5:**
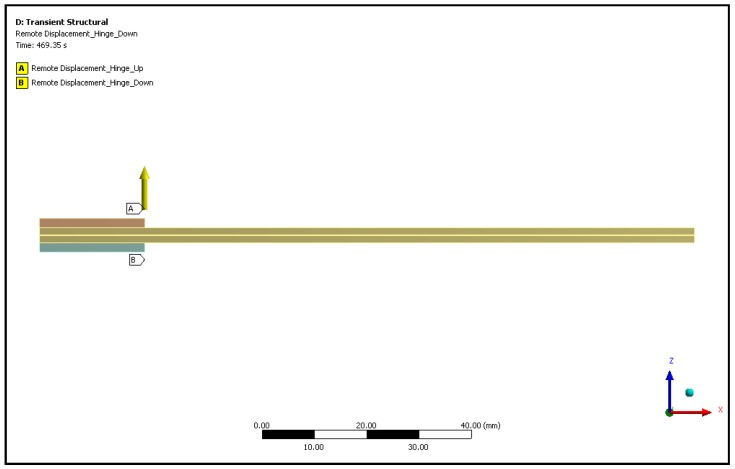
Applied load in the numerical model of the double cantilever beam (DCB) delamination tests.

**Figure 6:**
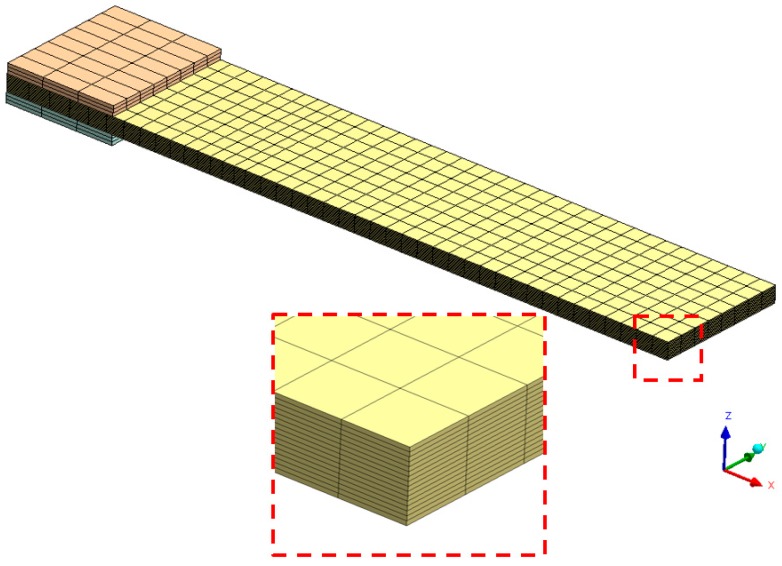
Mesh of the specimen in the numenrical model of the DCB delamination tests.

As far the actual tests are concerned, the numerical model (see Figure 5 and Figure 6) was representative of the flat hinges method [18] and the hinge rotations were taken into account by means of remote points (pilot nodes), connected to the inner edges of the plates, representing the part of the hinges glued to the specimen, in which the proper rotational degree of freedom was left free. 

**Figure 7:**
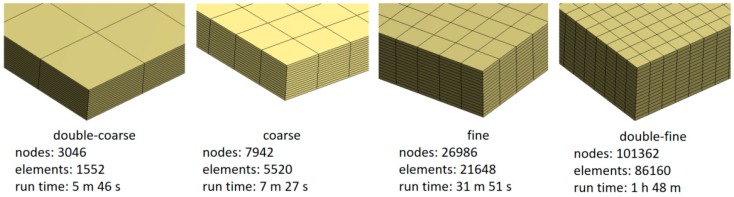
Mesh sensitivity analysis: mesh sizes.

**Figure 8:**
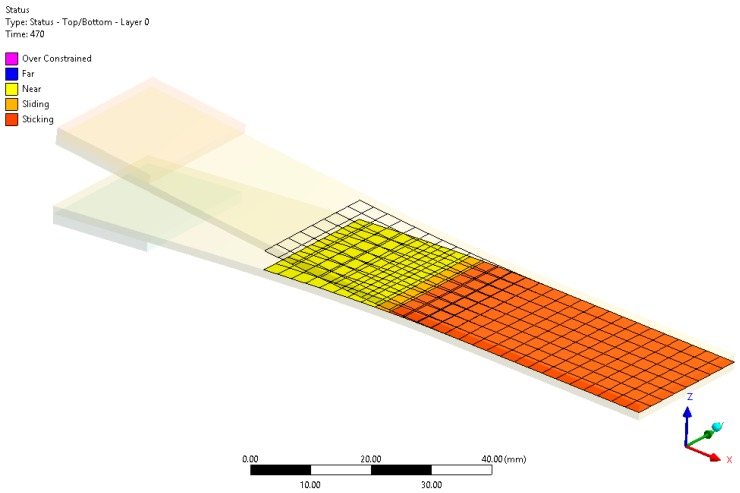
Contact elements and their status after flaw propagation.

The numerical model was built by means of solid elements (SOLID185 structural elements, CONTA173-TARGE170 contact elements for CZM method usage [36]), one layer of elements for each ply of the composite laminate, in order to guarantee enough accuracy in the calculation of the stress through the thickness of the sample (Figure 7). As far as the size of the mesh in the specimen’s plane is concerned, a mesh sensitivity analysis was carried out in order to find a trade-off between solution reliability and computational time. The aim of the FEA modeling was to calibrate the constitutive model by identifying the set of constants related to the CZM method (e.g. the delamination energy; maximum normal contact stress, also known as cohesive strength). In order to achieve this result, the optimization tool (i.e., modeFRONTIER) needs to compute FE models in a shorter time, because hundreds of runs (managed by the optimization algorhitms) are generally required to get a good fitting with the target curve. The mesh sensitivity analysis showed a behavior quite independent from the in-plane mesh size, because of the fact that both parts of the model (below and upside the delamination plane) had homologous meshes. This means that the positions of the nodes of both parts matched each other, nodes shared by the contact elements on which the CZM method was activated (Figure 8). During the analyses, no convergence issues arose, thus the default settings (L2-Norm) were mantained.

**Figure 9:**
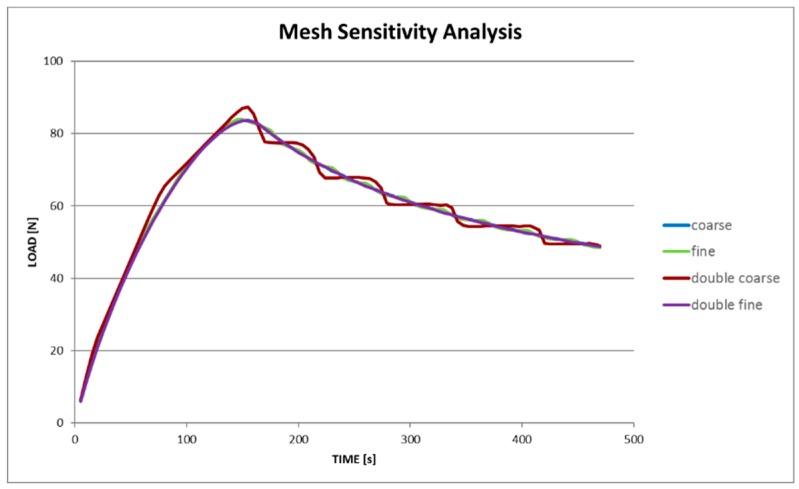
Mesh sensitivity analysis: result comparison.

The mesh sensitivity analysis was carried out by considering four kinds of mesh sizes, named “double-fine”, “fine”, “coarse”, and “double-coarse” (Figure 7). The performance comparison is reported in Figure 9. It demonstrates that, from the coarse model on, the results are pretty overlapped. For the calibration in modeFRONTIER, the coarse model was chosen. The analyses were carried out on a workstation esacore with 64 Gb of RAM.

**Figure 10:**
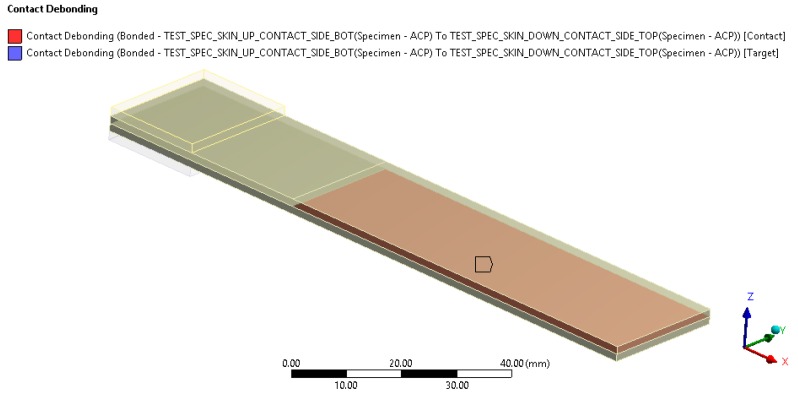
Contact debonding area in the specimen.

In Figure 10, the debonding area is highlighted. Following the recommended procedure for fracture mechanics, after the bonded contact set up, all cohesive properties were applied using fracture mechanics options in the highlighted area.

**Figure 11:**
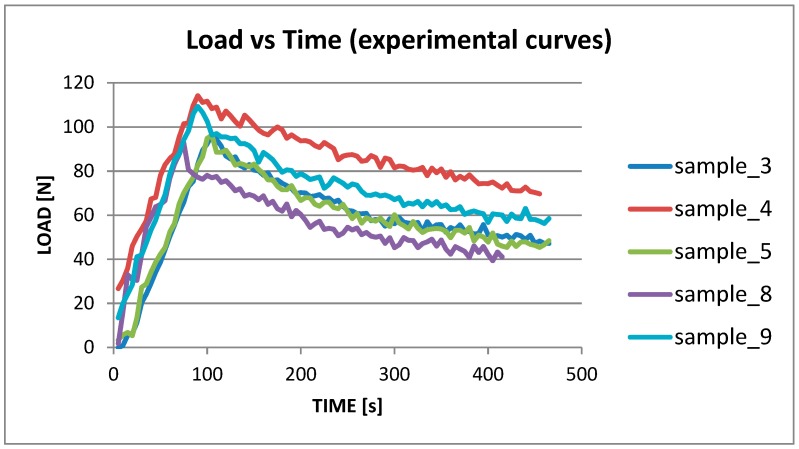
Experimental DCB curves for samples 3, 4, 5, 8, and 9.

In order to carry out the experimental/numerical calibration of the CZM model, it was necessary to identify idoneous comparison curves (load versus time) among the experimental ones; all the experimental curves were examined, but the tests in which a detachment of the hinges occured were disregarded. In such a way, the number of useful curves was reduced to five (related to specimens’ number 3, 4, 5, 8, and 9), and they are plotted in Figure 11. It can be seen that deformation energy is accumulated in the sample in a load range between 98 N and 115 N. In correspondence with those values, the accumulated energy (G_I_) reaches the critical value (G_IC_) so that the delamination starts. After that point, the material response becomes non-linear. The curves related to the samples 3 and 5 are quite overlapped and the related G_I_ energy values are close as well, a bit higher than the average value (about 11%). In Table 1, a synopsis of G_I_ energy values is reported for the different computation approaches [18].

**Figure 12:**
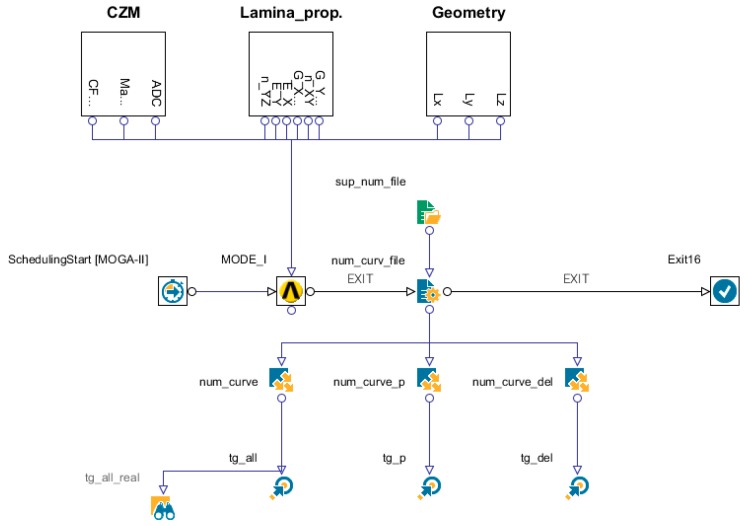
ModeFRONTIER workflow for the numerical/experimental calibration. CZM—cohesive zone material.

The curve related to specimen number 3 was chosen as a target. To take into account the possible influence of the adhesion force of the teflon insert (representing the initial defect as trigger of delamination), the FEM model was made parametric. The workflow of modeFRONTIER is reported in Figure 12. 

Two different types of DoEs (designs of experiment) and one optimization algorithm were used during the numerical experimental calibration with modeFRONTIER, in order to target the calibration in a fast and efficient way.

In particular, in different steps, the utilized DoEs are as follows [37]: 

Uniform Latin hypercube (ULH): this is a stochastic DoE algorithm that generates random numbers conforming to a uniform distribution. It is particularly suited for optimization with genetic algorithms and Response Surface Methodology training. ULH is an advanced form of Monte Carlo sampling; more precisely, it is constrained Monte Carlo sampling in which the constraint refer to the way each variable is sampled. The uniform statistical distribution is split in *n* intervals with the same probability and a random value is selected in each interval. ULH also tries to minimize correlations between input variables and to maximize the distance between the generated designs. In this way, the points are relatively uniformly distributed over the variable range. This algorithm is particularly suitable for generating the initial dataset for the optimization with a genetic algorithm and RSM training. 

Incremental space filler (ISF): this is an augmenting algorithm thta sequentially adds new design configurations to a database by maximizing the minimum distance from the existing points (optimization of the maximin criterion). This algorithm is particularly suited for generating a dataset for response surface training as it improves both the RSM approximation quality and reliability, as well as the numerical stability of the training. In general, new points are added in such way to uniformly fill the input space. However, if the zone filling option is enabled, the new points will be added in hyperspheres centered around the marked designs from the existing database and with a defined radius, expressed as a percentage of the variable ranges.

On the other hand, the optimization algorithm was MOGA-II (Multi-Objective Genetic Algorithm II) [37]. It is the ESTECO proprietary version of the multi-objective genetic algorithm that uses a smart and efficient multi-search elitism, which is able to preserve excellent (Pareto or non-dominated) solutions without converging prematurely to a local optimum. ESTECO is an independent software provider, highly specialized in numerical optimization and simulation data management with a sound scientific foundation and a flexible approach to customer needs. Elitism improves the convergence of the algorithm and ensures that the fitness of each new generation is greater than the fitness of the parent generation. The elitism operator works in the following way: 

The algorithm starts from an initial population, with an empty elite set, and uses the MOGA operators to generate offspring (next generation). Each new design is generated with one of the available operators.

The fitness of all individuals in the generation is computed to determine which are the best designs.

All non-dominated designs are copied and stored as the elite set. Each time this operation is performed and new designs are added to the elite set, duplicated and dominated designs are removed and the elite set is reduced to the population size by randomly removing designs in excess.

The next generation is computed by applying the MOGA operators and using the designs from the parent population (selected from the previous generation based on their fitness) and the elite set.

Steps 2–4 are iterated until the maximum number of generations is reached.

If elitism is not used, each next generation is computed only using the designs from the parent generation. MOGA-II handles constraints by applying the penalty policy. Error designs are always dominated by valid designs, whether feasible or unfeasible. Unfeasible designs can lie on the Pareto front only if there are no feasible designs. When a generation is complete, all its designs are taken into account, even error designs, for the computation of the new generation, ensuring the continuation of the optimization.

## 3. Results

In this work, the DCB samples were loaded by following the suggested requirements of ASTM D5528 [18], with the aim of investigating the delamination process occurring in carbon/epoxy composites subjected to mode I test.

**Figure 13:**
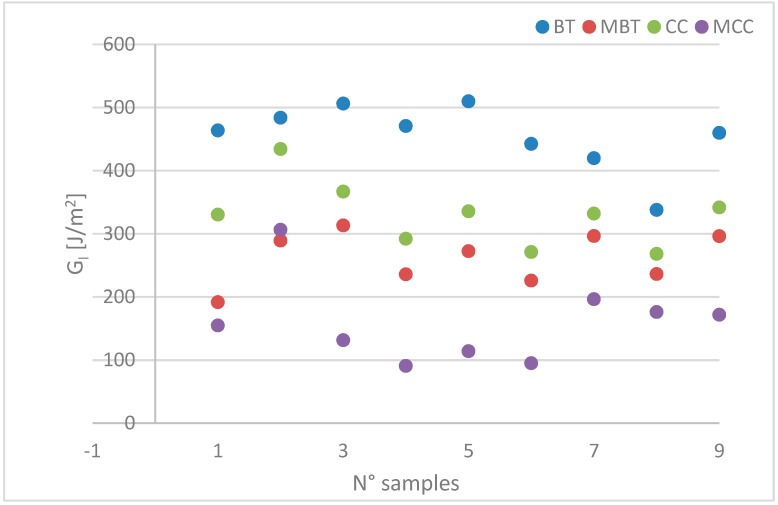
Comparison of the calculation methods for mode I delamination tests. BT—beam theory; MBT—modified BT; CC—compliance calibration; MCC—modified CC.

In Figure 13, a comparison among the four calculation methods for fracture toughness is reported. It should be noticed that the method based on BT provides the highest values of G_I_ with respect to the correct one (MBT), and both CC and MCC. This confirms that the BT hypothesis of ideal perfect constraint at the crack tip does not correspond to the real conditions of the specimens.

**Figure 14:**
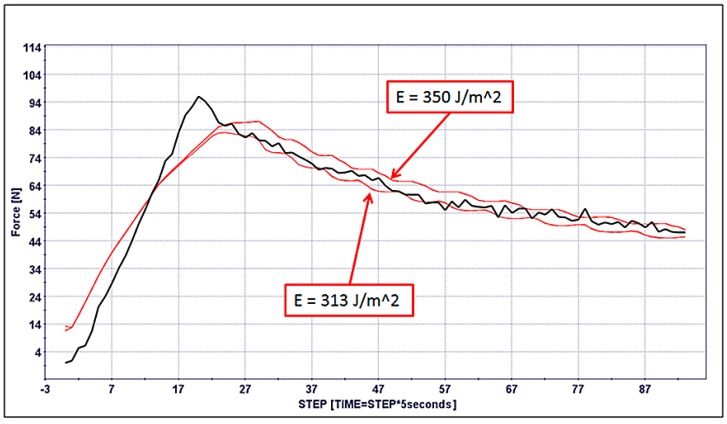
Comparison of the effect of the delamination energy G_I_ on the performances of the best designs. Black curve reports the experimental data of the specimen 3, while red ones are the numerical results with the two different values of energy.

The numerical/experimental calibration showed that the adhesion force of the Teflon does not affect the first part of the delamination curve, which is always characterized by a gap between the numerical data and the experimental data. The delamination was caught by the numerical model with a good approximation (without changing meaningfully the mechanical properties of the plies). The comparison of the experimental delamination energies (Table 2 and Table 3) suggested re-running the selected designs by assigning the experimental energy delamination of specimen 3 calculated by MBT, that is to say, 313 J/m^2^. The obtained result is depicted in Figure 14 and provides evidence that the calibration of the numerical model is robust in a range of ±5% with respect to the central value (330 J/m^2^) of the G_I_ energy delamination. 

## 4. Conclusions

In this paper, mode I delamination tests were carried out. The experimental tests were compared with the numerical model in order to validate the numerical results. The overlapping of the delamination curves was kept in good approximation by the numerical model. One specimen value for fracture toughness was taken into account for running the selected design. The value corresponds to the MBT and confirmed the robustness of the numerical calibration in modeling the real mechanical response of CFRP specimens.

## Figures and Tables

**Table 1:** Synoptic of the experimental G_I_ energy values for delamination propagation. BT—beam theory; MBT—modified BT; CC—compliance calibration; MCC—modified CC.

	M1_1	M1_2	M1_3	M1_4	M1_5	M1_6	M1_7	M1_8	M1_9
BT G_I_ [J/m^2^]	463.39	483.67	506.03	470.57	509.72	442.16	419.61	337.70	459.64
MBT G_I_ [J/m^2^]	191.42	289.06	313.01	235.64	272.26	225.60	296.17	235.98	295.99
CC G_I_ [J/m^2^]	330.18	434.04	366.57	291.97	335.33	270.95	331.72	267.96	341.57
MCC G_I_ [J/m^2^]	154.60	306.14	131.29	90.46	113.90	94.86	196.29	175.90	171.62

**Table 2:** Numerical delamination energies.

ID	E_X [Pa]	E_Y [Pa]	G_XY [Pa]	G_YZ [Pa]	n_XY	n_YZ	CF Energy [J/mm^2^]	E teflon [J/mm^2^]	Max_NC Stress [Pa]	Stress Teflon [Pa]
**271**	1.65 × 10^11^	8.5 × 10^9^	3.78 × 10^9^	2.63 × 10^9^	0.34	0.4	3.5 × 10^−4^	1.0 × 10^−6^	2.0 × 10^6^	2.0
**387**	1.65 × 10^11^	8.5 × 10^9^	3.78 × 10^9^	2.63 × 10^9^	0.34	0.4	3.5 × 10^−4^	3.0 × 10^−6^	2.0 × 10^6^	2.0
**456**	1.65 × 10^11^	8.5 × 10^9^	3.78 × 10^9^	2.63 × 10^9^	0.34	0.4	3.5 × 10^−4^	3.0 × 10^−6^	2.0 × 10^6^	6.0
**478**	1.65 × 10^11^	8.5 × 10^9^	3.78 × 10^9^	2.63 × 10^9^	0.34	0.4	3.5 × 10^−4^	3.0 × 10^−6^	2.0 × 10^6^	6.0

**Table 3:** Experimental delamination energies.

	M1_1	M1_2	M1_3	M1_4	M1_5	M1_6	M1_7	M1_8	M1_9
**BT GI [J/m^2^]**	463.39	483.67	506.03	470.57	509.72	442.16	419.61	337.70	459.64
**MBT GI [J/m^2^]**	191.42	289.06	313.01	235.64	272.26	225.60	296.17	235.98	295.99
**CC GI [J/m^2^]**	330.18	434.04	366.57	291.97	335.33	270.95	331.72	267.96	341.57
**MCC GI [J/m^2^]**	154.60	306.14	131.29	90.46	113.90	94.86	196.29	175.90	171.62
